# Beneficial factors for biomineralization by ureolytic bacterium *Sporosarcina pasteurii*

**DOI:** 10.1186/s12934-020-1281-z

**Published:** 2020-01-23

**Authors:** Liang Ma, Ai-Ping Pang, Yongsheng Luo, Xiaolin Lu, Fengming Lin

**Affiliations:** State Key Laboratory of Bioelectronics, School of Biological Science and Medical Engineering, Southeast University, Nanjing, 210096 Jiangsu People’s Republic of China

**Keywords:** *Sporosarcina pasteurii*, Biomineralization, Bacterial surface potential, Urease, ATP synthase

## Abstract

**Background:**

The ureolytic bacterium *Sporosarcina pasteurii* is well-known for its capability of microbially induced calcite precipitation (MICP), representing a great potential in constructional engineering and material applications. However, the molecular mechanism for its biomineralization remains unresolved, as few studies were carried out.

**Results:**

The addition of urea into the culture medium provided an alkaline environment that is suitable for *S*. *pasteurii.* As compared to *S. pasteurii* cultivated without urea*, S. pasteurii* grown with urea showed faster growth and urease production, better shape, more negative surface charge and higher biomineralization ability. To survive the unfavorable growth environment due to the absence of urea, *S. pasteurii* up-regulated the expression of genes involved in urease production, ATPase synthesis and flagella, possibly occupying resources that can be deployed for MICP. As compared to non-mineralizing bacteria, *S. pasteurii* exhibited more negative cell surface charge for binding calcium ions and more robust cell structure as nucleation sites. During MICP process, the genes for ATPase synthesis in *S. pasteurii* was up-regulated while genes for urease production were unchanged. Interestingly, genes involved in flagella were down-regulated during MICP, which might lead to poor mobility of *S. pasteurii.* Meanwhile, genes in fatty acid degradation pathway were inhibited to maintain the intact cell structure found in calcite precipitation. Both weak mobility and intact cell structure are advantageous for S*. pasteurii* to serve as nucleation sites during MICP.

**Conclusions:**

Four factors are demonstrated to benefit the super performance of *S. pasteurii* in MICP. First, the good correlation of biomass growth and urease production of *S. pasteurii* provides sufficient biomass and urease simultaneously for improved biomineralization. Second, the highly negative cell surface charge of *S. pasteurii* is good for binding calcium ions. Third, the robust cell structure and fourth, the weak mobility, are key for *S. pasteurii* to be nucleation sites during MICP.

## Background

Microbially induced calcite precipitation (MICP) refers to the formation of calcium carbonate in the presence of the different metabolic activities of microorganisms, including urealysis, photosynthesis, denitrification, ammonification, sulfate reduction and methane oxidation [1, 2]. It is widely used to repair cracks of constructions and roads, solidify sand and gravels, manufacture self-healing cement, develop bio-mineralization materials and so on [3]. Up to now, a large number of microorganisms have been discovered to perform MICP by ureolysis for various applications. For example, *Shewanella* has a positive effect on the compressive strength of mortar specimens [4]. A two-component self-healing bio-chemical agent was developed by mixing calcium lactate with spores of *Bacillus alkalinitrilicus* isolated from alkaline lake soil (Wadi Natrun, Egypt) to improve the durability of concrete structures [5]. Ureolytic bacterium *Pararhodobacter* sp. was used to solidify coral sand [6]. Among these well-known mineralizing bacteria, *S. pasteurii* was the most frequently used in MICP [7–9].

Under proper conditions, *S. pasteurii* can produce urease to decompose urea into carbonate and ammonia. The resulted carbonate reacts with calcium ions, forming the calcite (calcium carbonate) precipitation [10, 11]. *S. pasteurii*-mediated MICP has been widely applied in constructional engineering and bio-materials. The culture conditions of *S. pasteurii* were optimized to endow it with the strong ability of mineralization for hardening a sponge bar [12]. Through morphological observations, it was revealed that *S. pasteurii* could form a high degree of calcite crystals for bridging, coating on sand particles and infilling pore spaces, which was used to solidify sandy soil columns [13]. *S. pasteurii* and bacterially produced γ-polyglutamate (PGA) solution were applied to produce nacre-inspired layered calcium carbonate-polyglutamate composite materials that exhibited high extensibility and stiffness [14]. However, there are application limitations for *S. pasteurii*-mediated MICP including its vulnerability to different application conditions, uncontrolled growth, undesired by-products, and inadequate engineering scale-up. Some previous researches have tried to solve these problems by optimization of growth and mineralization conditions for *S. pasteurii*. The concentrations of urea, calcium chloride and nickel were optimized to maximize the growth rate and the calcium carbonate precipitation ability of *S. pasteurii* using the response surface methodology [15]. A combination of meat extract and sodium acetate was a suitable replacement for yeast extract in the growth medium for *S. pasteurii* with enhanced calcium carbonate formation in concrete by markedly reducing the retardation of cement hydration [16]. Furthermore, as *S. pasteurii* could not grow and synthesize urease anaerobically, repeated supply of cells and/or oxygenated medium might be necessary, especially when the ureolysis happens in areas far from the injection point [17]. In contrast to these extensive efforts on the practical applications of *S. pasteurii*-mediated MICP, few studies have been carried out to unravel the mechanism underlying biomineralization of *S. pasteurii*, which definitely would benefit its further practical applications.

In this study, we explored the potential processes involved in the biomineralization of *S. pasteurii.* The genome of *S. pasteurii* was sequenced and its cell surface charge was measured and compared to the non-mineralizing microorganism. The effect of urea was investigated on the biomass, the urease production, morphology, cell surface charge and the transcriptional level of *S. pasteurii,* and the pH of culture medium to demonstrate why *S. pasteurii* for MICP is generally grown with urea. Finally, the impact of MICP on the morphology and the transcription level of *S. pasteurii* was studied. This study sheds light on the molecular mechanism underlying the biomineralization of *S. pasteurii*, which will help to guide its application studies in the future.

## Results and discussion

### Genomic properties of *S. pasteurii* BNCC 337394

**Fig. 1:**
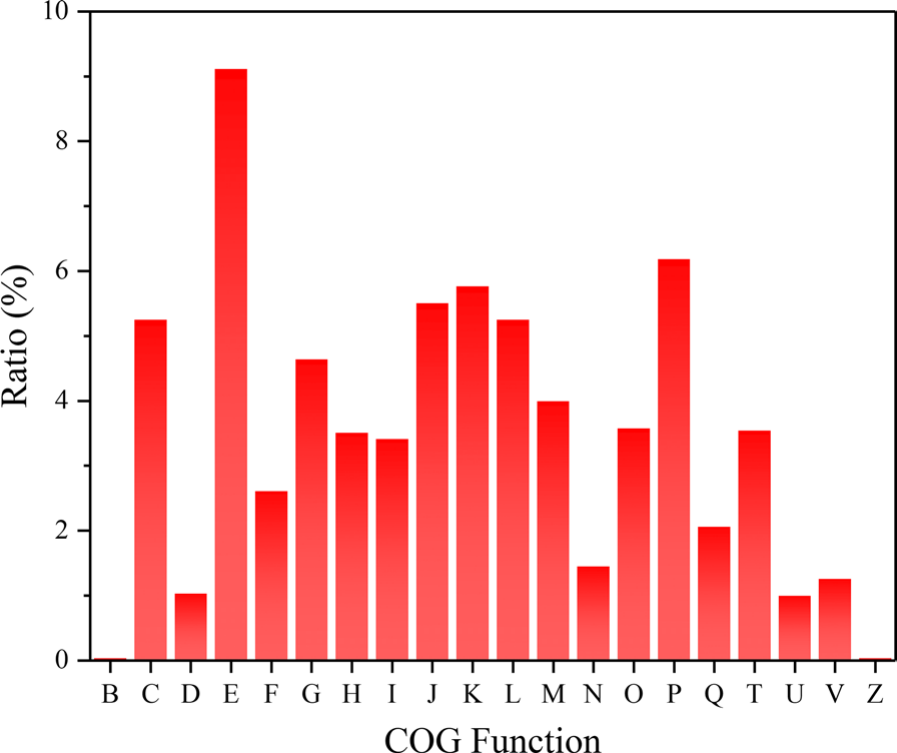
COG analysis of *S. pasteurii*. The vertical coordinate presented the ratio of the number of genes included in each classification to the total number of functional genes. COG designations are described as follows: B, chromatin structure and dynamics; C, energy production and conversion; D, cell cycle control, cell division, chromosome partitioning; E, amino acid transport and metabolism; F, nucleotide transport and metabolism; G, carbohydrate transport and metabolism; H, coenzyme transport and metabolism; I, lipid transport and metabolism; J, translation, ribosomal structure and biogenesis; K, transcription; L, replication, recombination and repair; M, cell wall/membrane/envelope biogenesis; N, cell motility; O, posttranslational modification, protein turnover, chaperones; P, inorganic ion transport and metabolism; Q, secondary metabolites biosynthesis, transport and catabolism; T, signal transduction mechanisms; U, intracellular trafficking, secretion, and vesicular transport; V, defense mechanisms; Z, cytoskeleton. This figure didn’t contain the genes of unknown function (S) and general function prediction only (R)

The genomic information of *S. pasteurii* plays an essential role in its further molecular mechanism study on bio-mineralization. Recently, *S. pasteurii* NCTC4822 was sequenced by whole genome shotgun with the NCBI Accession Number: NZ_UGYZ01000000 (https://www.ncbi.nlm.nih.gov/nuccore/NZ_UGYZ00000000.1). Its chromosome is 3.3 Mb, containing 3063 coding genes [18]. Nevertheless, *S. pasteurii* BNCC 337394 used in this study has not been sequenced. Thus, we performed the de-novo whole genome sequencing for *S. pasteurii* strain BNCC 337394, showing that its genome is 3.25 Mb with GC content of 39.17% and contains 3105 coding genes (Additional file 1), similar to that of strain NCTC4822. The COG analysis of the genome of *S. pasteurii* showed there were 2106 genes with known protein function and 999 genes whose protein functions were unknown (Additional file 2). The ratio of the number of genes in each classification to the total number of functional genes was calculated. Among the genes of known function, the ratio of genes related to amino acid transport and metabolism (E) was the largest, followed by genes involved in inorganic ion transport and metabolism (P) and transcription (K) (Fig. 1). *S. pasteurii* produces urease to convert urea to CO_3_^2−^ that reacts with Ca^2+^ to form CaCO_3_, which is the key to biomineralization [19]. Urease is composed by amino acids, requiring the functions such as Amino acid transport and metabolism (E), and Transcription (K). More particularly, through the Inorganic ion transport and metabolism (P), the inorganic ions nickel, a key part of the active center of urease, could regulate the urease activity to improve the ability of urealysis [20]. Therefore, the high proportion of functions E, P, and K in *S. pasteurii* might be beneficial for the action of urease, helping implement its mineralization ability.

Additionally, seven genes involved in urease assembly and activation were found in the sequenced genome of *S. pasteurii* as a gene cluster (Additional file 3: Table S1). UreA, ureB and ureC were three subunits of the main structure of urease holoprotein (UreABC)_3_, while ureD, ureF and ureG were three essential urease accessory proteins forming the complex UreDFG required for the functional incorporation of the urease nickel metallocenter to mature urease. One (UreABC)_3_ and three UreDFG form the (UreABC-UreDFG)_3_ complex. Then ureE as a nickel donor takes nickel ions into the enzyme active center of the complex to dissociate the accessory proteins in order to activate urease [21, 22]. As found by COG analysis, genes *ureA*, *ureB* and *ureC* belong to Amino acid transport and metabolism (E), while *ureD*, *ureE* and *ureF* are assigned to Post-translational modification, protein turnover and chaperones (O). Gene *ureG* is attributed to both Transcription (K) and Post-translational modification, protein turnover and chaperones (O). These urease-related genes can serve as potential targets by metabolic engineering to improve the efficiency of urease production in *S. pasteurii* for enhanced bio-mineralization in future study.

### The effect of urea in culture medium on *S. pasteurii*

**Fig. 2:**
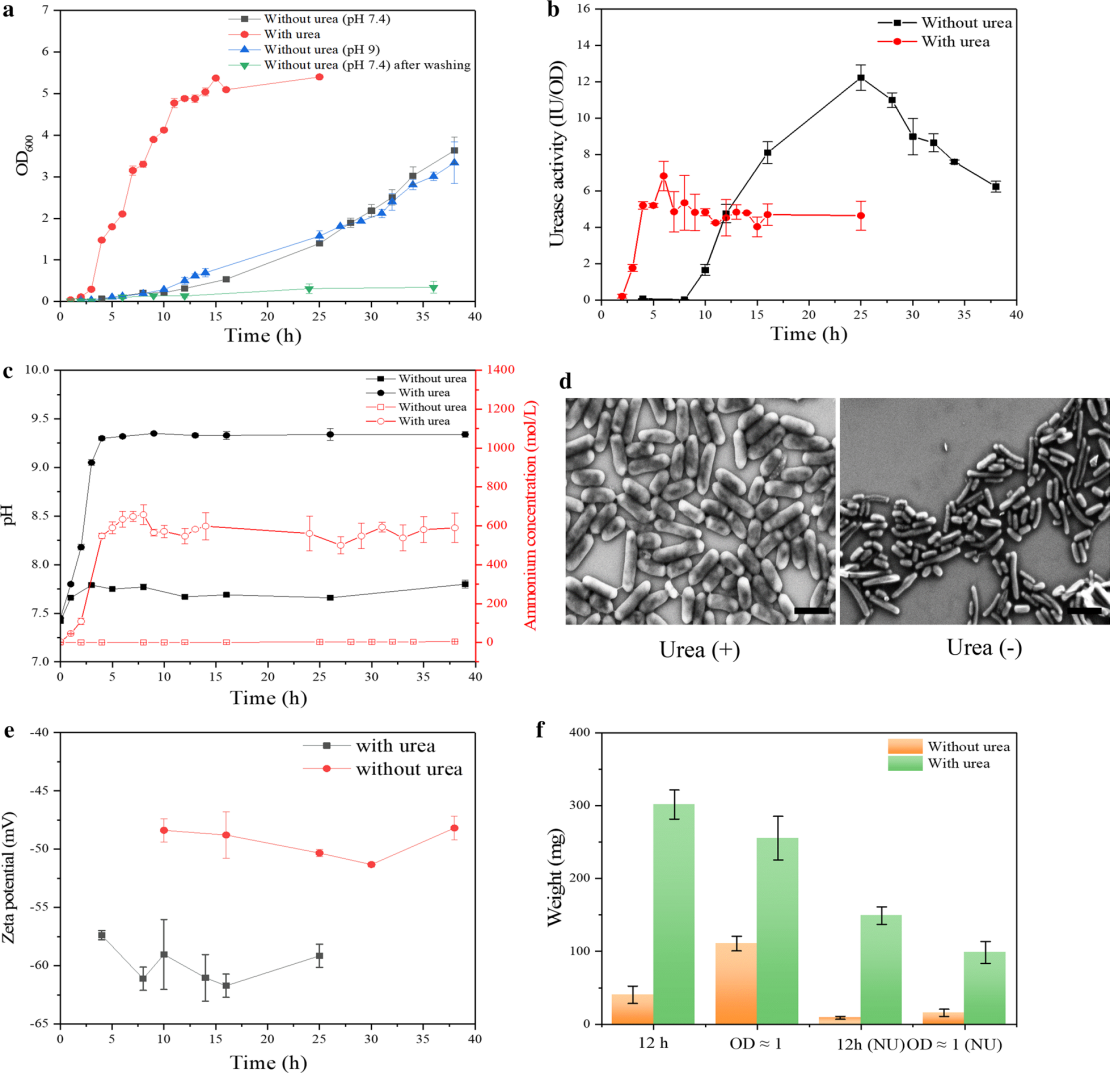
**a** The bacterial growth of *S. pasteurii* cultivated TSB with urea (red), and without urea at pH 7.4 (black), and without urea at pH 9.0 (blue) in 40 h were measured. With the removal of residue urea in the preculture medium, the bacterial growth of *S. pasteurii* is showed in green line. The intracellular urease activity **b**, ammonium concentration (**c**, red line) and pH of bacterial suspension (**b**, black line) of *S. pasteurii* cultivated with urea and without urea in 40 h were measured. The morphology of *S. pasteurii* cultivated with urea (+) and without urea (−) **d** was observed by SEM. The trend of zeta potential of *S. pasteurii* cultivated with urea was compared to that without urea **e** at different time points. All scale bars are 2 μm. The weight of precipitation produced by *S. pasteurii* cultivated with urea and without urea at 12 h or at OD_600_ = 1 **f** were compared. NU meant that no extra urea was added during the process of mineralization

We investigated the effect of urea, a key component of the bio-mineralization, on the growth, intracellular urease activity, morphology, cell surface charge and mineralization ability of *S. pasteurii* grown in TSB medium. With urea, the optical density (OD) of *S. pasteurii* at 600 nm was increased sharply to OD_600_ ≈ 5 within 12 h and then entered a steady period (Fig. 2a). Accordingly, its enzyme activity had a rapid increase to the maximal 6.8 IU/OD at 6 h with a slight decrease to 4.5 IU/OD before reaching the plateau (Fig. 2b). Without urea, the OD_600_ of *S. pasteurii* was only 0.31 at 12 h, much lower than that with urea (OD_600_ ≈ 5), and reached 3.6 at 38 h (Fig. 2a). Meanwhile, its enzyme activity did not increase observably until 10 h. The maximal enzyme activity was about 12 IU/OD at 25 h, exceeding that (6.8 IU/OD) in the presence of urea at 6 h (Fig. 2b). Obviously, *S. pasteurii* cultivated with urea showed faster growth and urease production than that without urea, though its maximal urease activity was much lower. This finding was consistent with previous studies that revealed the urease synthesis was repressed in the presence of ammonia in *Klebsiella aerogenes*, *Bacillus subtilis* and *Rhizobium meliloti* [23–25]. TSB itself did not produce ammonium, while the addition of urea can generate a high level of ammonium (Fig. 2c), inhibiting the production of the urease synthesis in *S. pasteurii*.

During the growth of *S. pasteurii* with urea, the generated urease quickly converted urea into ammonium (Fig. 2c), making the pH of culture medium increase rapidly from 7.4 to 9.4 in 5 h (Fig. 2c), accompanied by the same upward trend of ammonium ions. The resulted alkaline environment is suitable for the proliferation of *S. pasteurii*, an alkalophilic bacterium. By contrast, the medium without urea kept at pH 7.7 throughout the whole growth phase due to the unavailable ammonium (Fig. 2c), which was not suitable for the growth of *S. pasteurii*, resulting in slow proliferation. As observed by scanning electron microscopy (SEM), *S. pasteurii* cultivated with urea was health with intact cells (Fig. 2d), while *S. pasteurii* cultivated without urea became out of shape and slightly maldeveloped (Fig. 2d). Besides, *S. pasteurii* cultivated with urea displayed more negative charge (− 57 to − 63 mV) than that cultivated without urea (− 48 to − 53 mV) during the whole growth process (Fig. 2e).

The weight of precipitation produced by the bacterial suspension of *S. pasteurii* grown with and without urea was analyzed either with the same growth OD_600_ (OD_600_ ≈ 1) where the urease activity was ~ 5.6 IU/OD with urea, and ~ 11.6 IU/OD without urea or at the same growth time (12 h). In both cases, the weight of precipitation produced by *S. pasteurii* cultivated with urea was larger than that produced by *S. pasteurii* without urea (Fig. 2f). Even when there was no extra urea added for mineralization, *S. pasteurii* cultivated with urea still produced more precipitation than that cultivated without urea (Fig. 2f). In the absence of urea culture, with extra urea, the precipitation produced at OD_600_ ≈ 1 was about 100 mg, more than that at 12 h because the urease activity at OD_600_ ≈ 1 was about 11.6 IU/OD, bigger than about 5 IU/OD at 12 h (Fig. 2b), which meant that high urease activity led to producing more CO_3_^2−^ for CaCO_3_ formation. While without extra urea, there was little precipitation comprised by bacterial cells at both OD_600_ ≈ 1 and 12 h, which indicated that the mineralization process of *S. pasteurii* mainly depended on urea hydrolysis. The mineralization of *S. pasteurii* worked best when grown in the presence of urea for 12 h where both OD_600_ and the intracellular urease activity of *S. pasteurii* reached a plateau, which was not achieved by other conditions tested here. The presence of urea enabled a good correlation of biomass growth and urease production of *S. pasteurii*, which is beneficial for its biomineralization.

Taken together, *S. pasteurii* grown in the presence of urea could simultaneously proliferate and produce urease more rapidly with a healthier cell form and more negative cell surface charge in an alkaline environment as compared to that grown in the absence of urea, showing significantly enhanced mineralization ability. That *S. pasteurii* was grown slowly in TSB without urea contradicted the early finding that *S. pasteurii* exhibited no growth in ordinary neutral media without urea supplementation [26]. We have grown *S. pasteurii* in TSB + 2% urea for pre-culture. Pre-cultures were diluted 1:100 into 100 mL TSB in 500-mL conical flasks. Although no extra urea was supplied, there might be a trace amount of urea or its degraded product ammonium from preculture, enabling *S. pasteurii* to grow in TSB without urea. To prove this, we have washed the preculture with PBS three times to remove the residue urea before inoculation into 100 mL TSB. With the removal of residue urea, *S. pasteurii* was not grown out at all in TSB without urea (Fig. 2a) as found in the previous study [26], evidencing our speculation.

### The effect of urea on the transcriptome of *S. pasteurii*

**Fig. 3:**
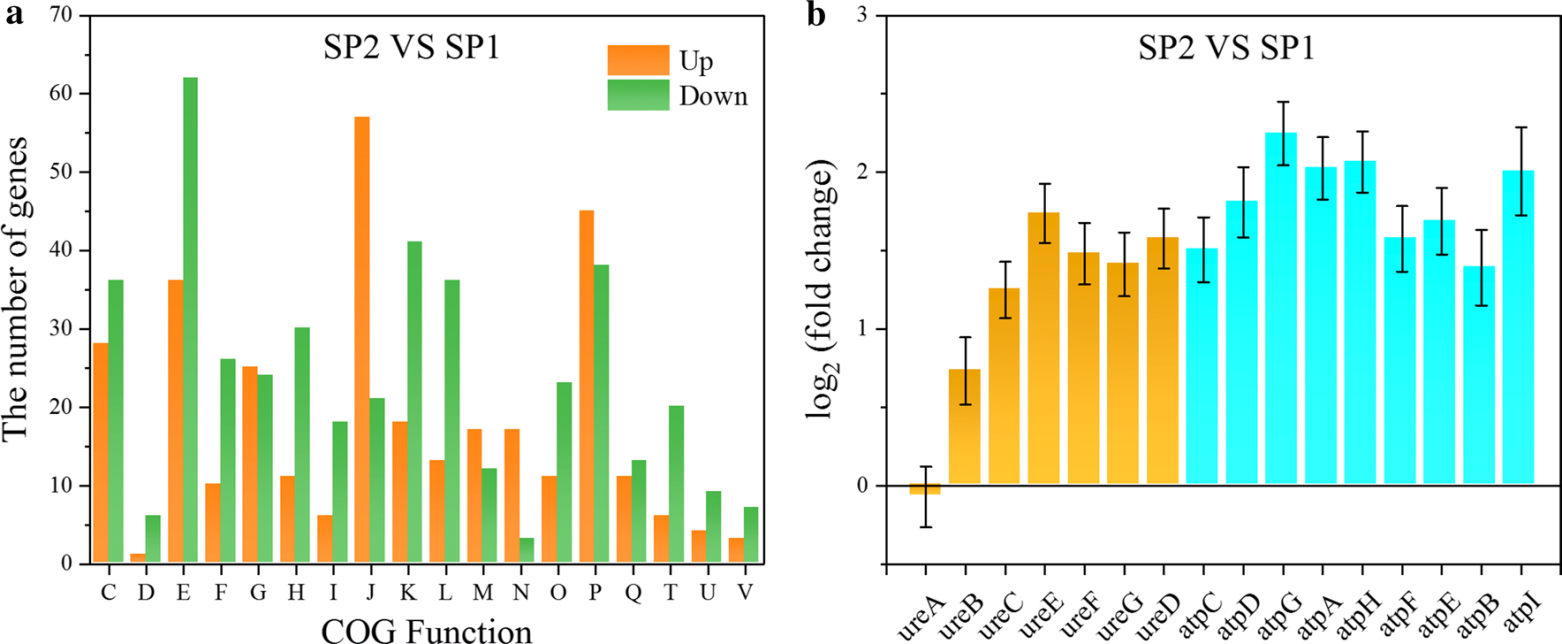
COG functions of all significant DEGs (**a**) and the relative transcription levels of genes related to urease and ATPase (**b**) are shown by comparing *S. pasteurii* without urea (SP2) to with urea (SP1)

To understand how urea influences *S. pasteurii* at the transcriptional level, we performed the transcriptome analysis of *S. pasteurii* cultivated in TSB medium with or without urea at the same OD_600_ ≈ 1, at which the corresponding intracellular urease activity was ~ 5.6 IU/OD and ~ 11.6 IU/OD, respectively. The total reads of *S. pasteurii* cultivated with urea were mapped to the reference genome (as we sequenced above) with coverage of ~ 98.15–98.44%. Genes were differentially expressed when the average reads of the corresponding transcripts differed with log_2_ (fold change) ≥ 1 and padj ≤ 0.05. By comparing *S. pasteurii* cultivated without urea (SP2) to with urea (SP1), there were a total of 1008 significant differentially expressed genes (DEGs) in the absence of urea, of which 421 were up-regulated and 587 down-regulated (Additional file 4). The DEGs were classified according to their gene functions (Fig. 3a). Except functions Translation, Ribosomal structure and biogenesis (J), Inorganic ion transport and metabolism (P) and Cell motility (N), most gene functions contained relatively more down-regulation genes, such as Energy production and conversion (C), Amino acid transport and metabolism (E) and Transcription (K).

Specifically, genes *ureD*, *ureE*, *ureF* and *ureG* that adjust the nickel active center and mature urease, and *ureC* for the urease structure assembly were notably increased, while *ureA* and *ureB* for the urease structure assembly stayed unchanged (Fig. 3a). Meanwhile, 41 out of 135 ribosomal genes were up-regulated DEGs (Additional file 3: Table S2). The up-regulation of genes involved in urease synthesis and ribosomal genes might be linked with the higher urease activity in *S. pasteurii* cultivated without urea as we observed (Fig. 2b).

The genes of F-type ATPase are all DEGs that were significantly up-regulated (Fig. 3b). F-type ATPase is divided into two regions F_1_ (containing genes *atpA*, *atpD*, *atpG*, *atpH*, and *atpC*) and F_0_ (containing genes *atpB*, *atpF*, and *atpE*). The F_1_ region is responsible for ATP hydrolysis, while the F_0_ region for proton transfer [27, 28]. Similar to urease genes, all these genes were also clustered in the genome of *S. pasteurii* (Additional file 3: Table S1). The ATP generation in *S. pasteurii* was coupled with urea hydrolysis At pH 9.4 optimum for *S. pasteurii growth*, the extracellular proton concentration was lower than the intracellular one, leading to a reversed ∆pH (the transmembrane pH gradient) with the tendency for protons to move from inside to outside the cells. To combat this, *S. pasteurii* have developed two alternative ways to increase proton motive force and drive protons into the cells for ATP generation: 1) alkalinisation of the cytoplasm, leading to a reduced ∆pH 2) efflux of other cations than H^+^, giving rise to an increased ∆ψ (membrane potential). If ∆ψ is large enough, proton motive force becomes sufficient to drive the influx of protons into the cells against the concentration gradient for ATP generation. Urea diffuses into bacterial cells according to concentration gradient, where it is hydrolyzed by urease to ammonium and carbonate, leading to the alkalinisation of the cytoplasm and a decreased ∆pH. Ammonium was excreted outside the cells according to its concentration gradient, causing an increased ∆ψ. The increased ∆ψ is reversed through driving protons into the cells against the concentration gradient, resulting in ATP generation [29–31]. This coupling was probably broken in the absence of urea as indicated by the slow growth of *S. pasteurii* in the absence of urea no matter the pH was optimum or not (Fig. 2a), leading to the lack of ATP. To supply enough ATP, the efficiency of ATPase can be improved by up-regulating the expression of ATPase genes.

14 genes related to flagella were significantly up-regulated and only 1 gene down-regulated (Table 1). Among these genes, *FliD* and *FliC* are related to the flagella filament that acts as a helical screw to produce thrust for swimming motility. *FliN*, *FliM* and *FliG* are responsible for the C ring structure acting not only as a central part of the rotor for torque generation but also as a structural device to switch the direction of motor rotation. *FliP*, *FliO*, *FliI* and *FliH* constitute the type III protein export apparatus that is responsible for transporting axial component proteins from the cytoplasm to the distal end of the growing flagella structure to construct the axial structure beyond the cellular membranes. *FliK* is related to hook structure to control the kook length and *FlgG* is related to the distal rod. Both *FliK* and *FlgG* are involved in regulating the bending flexibility of the hook and rod structure [32, 33]. Except *FlgG*, all genes were up-regulated in the absence of urea. The increased expression of these genes might allow for enhanced motility of *S. pasteurii* to approach the nutrients to survive the nitrogen starvation caused by the absence of urea from the culture medium [34]. However, it is north noting that *FlgG* was reduced. Why the expression of *FlgG* was downregulated specifically in the absence of urea and how it is going to affect the efficiency and function of flagella is unknown.

**Table 1:** DEGs related to flagellar proteins. The value was got by comparing the transcription level of *S. pasteurii* without urea to that with urea

GeneID^a^	GeneName	Log_2_ (fold change)^b^ (no urea/urea)	Padj^c^	Regulation
1_1693	FliP	1.46	5.57E−09	Up
1_1694	FliO	1.54	2.06E−11	Up
1_1696	FliN	1.97	3.87E−20	Up
1_1697	FliM	1.11	7.39E−08	Up
1_1702	FlgD	1.33	3.50E−07	Up
1_1703	FliK	1.69	3.62E−10	Up
1_1705	FliJ	2.16	2.31E−12	Up
1_1706	FliI	1.73	1.04E−13	Up
1_1707	FliH	1.83	7.02E−11	Up
1_1708	FliG	1.12	0.000114	Up
1_1955	FliT	2.37	3.83E−27	Up
1_1956	FliS	2.32	1.39E−24	Up
1_1957	FliD	1.62	1.84E−13	Up
1_1961	FliC	1.56	1.35E−08	Up
1_2278	FlgG	-1.93	7.37E−21	Down

^a^Gene_ID is the serial number of genes in the sequencing process

^b^log_2_ (fold change): the log base 2 of the variation

^c^padj: P-value adjusted by FDR (false discovery rate)

In summary, *S. pasteurii* cultivated in the absence of urea notably up-regulated the genes involved in urease synthesis, ATP synthesis and flagella to cope with the unfavorable culture conditions due to the absence of urea, which might compromise its biomineralization ability. The presence of urea provided *S. pasteurii* favorable culture conditions, saving extra energy to make it more prepared for biomineralization.

### *S. pasteurii* displays high negative surface charge

**Fig. 4:**
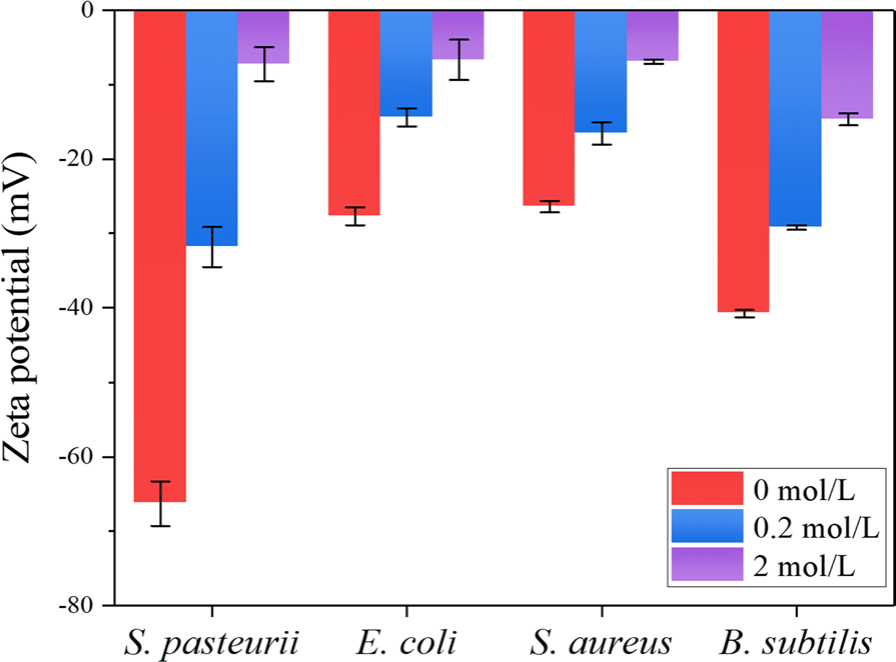
Zeta potentials of *S. pasteurii*, *E. coli*, *S. aureus* and *B. subtilis* cultivated in TSB medium with 20 g/L urea were tested with 0 mol/L, 0.2 mol/L and 2 mol/L CaCl_2_, respectively

In MICP, cationic calcium ions need to bind to the negatively charged surface of biomineralization microorganism, suggesting the microbial surface charge might play a role in the biomineralization capability of *S. pasteurii* [35]. Therefore, we measured the zeta potential of *S. pasteurii,* and non-mineralizing bacteria *Escherichia coli, Staphylococcus aureus* and *Bacillus subtilis*, which were cultivated in TSB with 20 g/L urea (Fig. 4). The potential of *S*. *pasteurii* was − 67 mV, while the other three non-mineralizing bacteria *E*. *coli*, *S*. *aureus* and *B*. *subtilis* were − 28, − 26 and − 40.8 mV, respectively. Obviously, *S*. *pasteurii* exhibited much more negative surface charge than non-mineralizing bacteria *E. coli, S. aureus* and *B. subtilis.* When treated with 0.2 mol/L or 2 mol/L CaCl_2_, a noticeable upward trend of the zeta potentials was found for all the tested bacteria with the largest increase observed for *S. pasteurii*. As we know, bacterial surface has various components contributing to negative charge such as phosphate groups, carbonate groups and sulfate groups. The metal ions like calcium, magnesium and copper can bind to them. That *S. pasteurii* has much more negative surface charge than other non-mineralizing bacteria indicates that there might be more negative functional groups on its surface for binding more metal ions like calcium ions in the same ionic environment. Even without urea, the negative charge of *S. pasteurii* was more than that of non-mineralizing bacteria *E. coli, S. aureus* and *B. subtilis* (Fig. 2e), indicating that the superb negative charge of S*. pasteurii* is constitutive which might be due to the components of the cell membrane. This can greatly benefit its mineralization process. It has been reported that the zeta potential of S*. pasteurii* ATCC 6453 and BNCC 337394 was − 19.51 to  − 23.10 mV and − 32.5 ± 1.3 to −35.6 mV respectively, higher than the zeta potential of S*. pasteurii* BNCC 337394 in this study [35, 36]. These distinctive zeta potential values might be attributed to the different strains, different culture mediums and/or different measurement conditions used in each study.

### *S. pasteurii* displayed intact cell structure under the biomineralization conditions

**Fig. 5:**
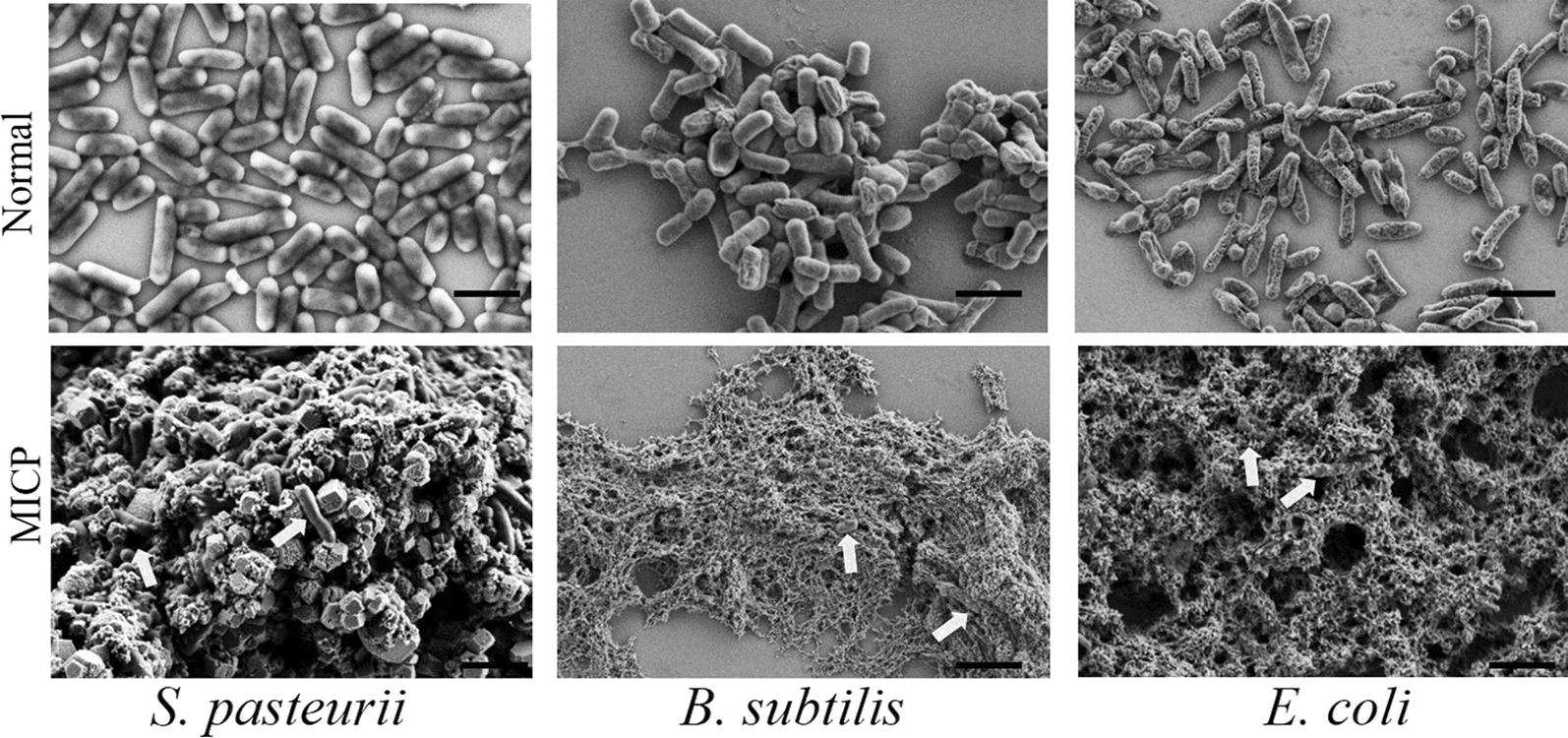
The SEM images of *S. pasteurii*, *B. subtilis* and *E. coli* cultivated in TSB with 20 g/L urea (Normal), and treated with 1 mol/L CaCl_2_ and 1 mol/L urea (MICP). The white arrows in the figures show the bacterial cells. All scale bars are 2 μm

It is reported that *S. pasteurii* serves as the nucleation sites in MICP for CaCO_3_ precipitation [37–39], requiring that the structure of *S. pasteurii* must be robust without lysis and maintain highly intact under the conditions of MICP. To prove this speculation, we measured the morphology of *S. pasteurii*, *B. subtilis* and *E. coli* after MICP by SEM (Fig. 5)*. S. pasteurii* grown in TSB medium with urea had perfectly intact cell forms. After being incubated with both CaCl_2_ and urea, the shape of *S. pasteurii* was complete and embedded inside lumpy substances (Fig. 5). The lumpy substances contained 11.26% calcium, 26.86% carbon, and 61.67% oxygen (Additional file 3: Table S3). The molar ratio of the three elemental components was close to 1:1:3, demonstrating that these substances were mainly calcium carbonate (CaCO_3_). Obviously, *S. pasteurii* maintained a highly intact cell structure even under mineralization condition. The non-mineralizing *B. subtilis* is supposed to share similar bacterial cell wall as *S. pasteurii*, for both of them are Gram-positive bacteria. However, *B. subtilis* was less healthy in TSB medium with urea than *S. pasteurii*. In the presence of CaCl_2_ and urea, few malformed *B. subtilis* were observed among the massive aggregates with an amorphous loose porous structure, indicating that most of *B. subtilis* cells had lysed. Gram-negative bacterium *E. coli,* another non-mineralizing bacterium, also behaved similarly to *B. subtilis* under the MICP conditions. Those amorphous loose porous matters that appear in association with *E. coli* and *B. subtilis* were probably CaCl_2_ and urea salts in addition to bacterial remains. According to the experimental conditions, only CaCl_2_ and urea were added to the bacterial cells. For SEM, the samples were dehydrated and dried, resulting in the precipitation of CaCl_2_ and urea.

Collectively, we found that *S. pasteurii* were grown healthier in slightly alkaline medium than non-mineralizing bacteria *E. coli* and *B. subtilis*. Under mineralization condition, *S. pasteurii* was still in great shape, even being surrounded by the compact calcium carbonate precipitation. By contrast, *B. subtilis* and *E. coli* became damaged and lysed inside amorphous loose porous aggregate (Fig. 5). Seemly, the robust structure of *S. pasteurii* under the mineralization conditions play an important role in its superior biomineralization ability in addition to its highly negative surface charge as we found above.

### Transcriptome analysis of *S. pasteurii* before and after mineralization

**Fig. 6:**
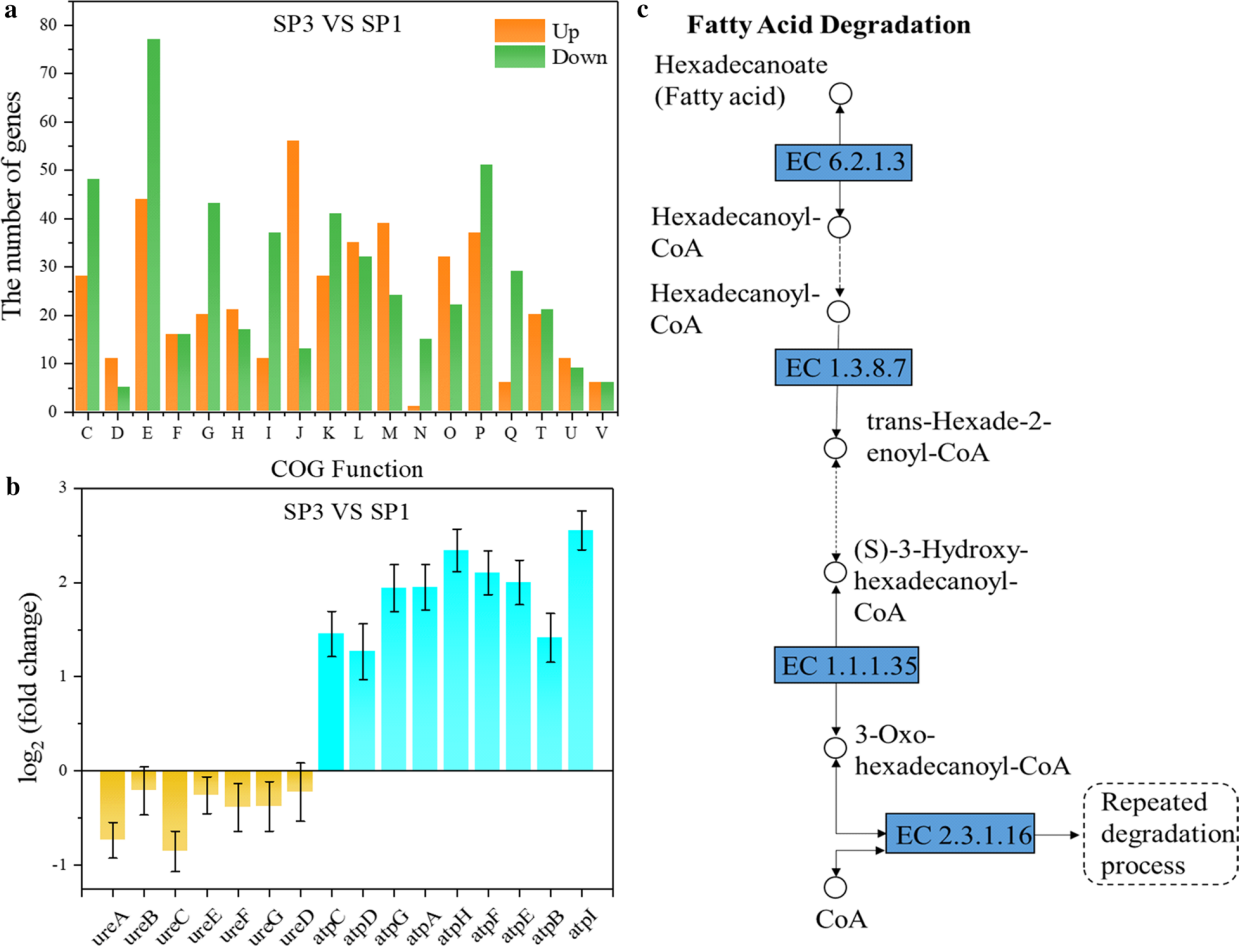
COG functions of all significant DEGs (**a**) and the relative transcription levels of genes related to urease and ATPase (**b**) are shown by comparing *S. pasteurii* after mineralization (SP3) to that before (SP1). The down-regulated expression of enzymes marked in blue are presented in the pathway of fatty acid degradation (**c**)

To understand how MICP influences *S. pasteurii* at the transcriptional level, transcriptome analysis was performed by comparing the transcriptional level of *S. pasteurii* after mineralization (SP3) with that before mineralization (SP1). The total reads of *S. pasteurii* were mapped to the reference genome (as we sequenced above) with coverage of ~ 80.42–89.47%. A total of 1316 DEGs of which 590 were up-regulated and 726 down-regulated were found after mineralization and classified (Additional file 4). Functions Cell cycle control, cell division, chromosome partitioning (D), Translation, ribosomal structure and biogenesis (J), Cell wall/membrane/envelope biogenesis (M) and Posttranslational modification, protein turnover, chaperones (O) contained obviously more up-regulated genes (Fig. 6a). The number of the up-regulated genes in Carbohydrate transport and metabolism (G) was similar to that of the down-regulated genes. Except those functions, the other functions contained more down-regulated genes.

During MICP, the genes related to ATPase of *S. pasteurii* were significantly up-regulated (Fig. 6b), whereas the genes of urease showed relatively unchanged expressions (Fig. 6b). We found that pH of *S. pasteurii* solution decreased from 9.31 to 8.22 during the mineralization process, which was not favorable for the coupling of ATP synthesis with urea hydrolysis, and the growth of *S. pasteurii*. Similar to the case without urea, *S. pasteurii* might increase the expression of genes encoding ATPase to keep supplying enough energy in unfavorable environment. In contrast, urease produced in sufficient quantities before mineralization might be stable enough that *S. pasteurii* did not need to enhance the expression of the related genes during MICP. It is demonstrated that the mineralization conditions promote the ATP synthesis of *S. pasteurii* but have no significant influence on urease production and activity.

The mRNA levels of genes encoding four key enzymes in fatty acid degradation pathway including long-chain acyl-CoA synthetase (EC 6.2.1.3), acyl-CoA dehydrogenase (EC 1.3.8.7), 3-hydroxyacyl-CoA dehydrogenase (EC 1.1.1.35) and acetyl-CoA C-acetyltransferase (EC 2.3.1.16) were significantly decreased under the mineralization condition (Fig. 6c and Additional file 3: Table S4). As fatty acids are essential components of cell membrane, the down-regulation of gene expression in fatty acid degradation pathway might partly explain why *S. pasteurii* can maintain an intact cell structure during MICP to serve as the nucleation sites.

Besides, it was interesting to observe that the gene expressions of flagellar proteins included in function cell motility (N) were almost inhibited (Table 2). These proteins are components of bacterial flagellum, which are involved, in bacterial motility and chemotaxis as mentioned above [32, 33, 40]. The decreased expression of the corresponding proteins in the bacterial flagellum of *S. pasteurii* might result in the inhibition of bacterial motility under mineralization conditions, which is beneficial for the biomineralization, giving that *S. pasteurii* cells serve as the nucleation sites during bacterial calcium precipitation. As compared to that without urea, the mRNA level of genes related to bacterial flagellum in *S. pasteurii* cultivated with urea was significantly reduced (Table 1), which was further decreased after mineralization (Table 2). Hence, we conjecture that the activity of bacterial flagellum of *S. pasteurii* might be closely related to the urea concentration in the environment. But more studies are required to confirm this hypothesis in the future.

**Table 2:** DEGs related to flagellar proteins by comparing the transcription level of *S. pasteurii* after mineralization to that before mineralization

GeneID	GeneName	Log_2_ (fold change) (MICP after/before)	Padj	Regulation
1_1709	FliF	− 1.54	9.94E−08	Down
1_1710	FliE	− 2.90	1.50E−16	Down
1_1711	FlgC	− 2.94	3.95E−16	Down
1_1712	FlgB	− 2.73	6.61E−12	Down
1_1955	FliT	− 1.07	0.000761	Down
1_1956	FliS	− 1.27	0.000319	Down
1_1957	FliD	− 1.81	8.88E−17	Down
1_1958	FlaG	− 2.17	2.93E−06	Down
1_1961	FliC	− 4.12	4.81E−42	Down
1_1966	FlgK	− 1.90	4.37E−14	Down
1_1692	FliQ	− 1.28	0.011284	Down

## Conclusions

In this study, the genome of *S. pasteurii* BNCC 337394 was sequenced. Urea in cultural medium was rapidly converted to ammonium, increasing pH of the culture medium from 7.4 to 9.4 and creating an alkaline environment that is suitable for the proliferation of *S. pasteurii*. As a result, the growth and urease production in *S. pasteurii* were accelerated notably at the same time, providing sufficient biomass and urease for biomineralization. Moreover, *S. pasteurii* cultivated with urea displayed healthier cell surface for nucleation sites and more negative cell surface charge for calcium ion binding than that without urea. All these led to improved biomineralization of *S. pasteurii* grown on urea, as compared to that of *S. pasteurii* grown without urea. To survive the unfavorable environment caused by the absence of urea, *S. pasteurii* increased the expression of genes involved in urease production, ATPase synthesis and flagella, which might occupy the resources for biomineralization. It was first reported that *S. pasteurii* displays superb negative charge, much more negative than that of non-mineralizing bacteria *E. coli, S. aureus* and *B. subtilis.* During MICP, *S. pasteurii* increased the mRNA levels of ATPase synthesis genes, while no impact was observed on the expression of urease genes. Genes related to flagella were down-regulated during MICP, which might cause weak mobility of *S. pasteurii.* More interestingly, the expression of genes responsible for fatty acid degradation was remarkably inhibited, suggesting that fatty acid degradation pathway might be shut down. This finding was consistent with the intact cell structure of *S. pasteurii* during MICP that was not seen for non-mineralizing bacteria. Both poor mobility and intact cell structure of *S. pasteurii* are favorable for its role as nucleation sites, contributing to its excellent biomineralization. These outcomes reveal the important factors for the super mineralization ability of *S. pasteurii*.

## Materials and methods

### Bacterial strains and growth conditions

Deionized water was obtained from a Milli-Q synthesis system (Billerica, MA, USA). Calcium chloride and urea were purchased from Sigma-Aldirch (St. Louis, Mo, USA). Phosphate buffer saline (PBS) (pH ≈ 7.4) was prepared with NaCl, KCl, Na_2_HPO_4_ and KH_2_PO_4_ purchased from Sigma-Aldirch (St. Louis, Mo, USA). *S. pasteurii* (BNCC 337394) was grown in trypticase soy broth (TSB) (Hope Bio-Technology Co., Ltd., Qingdao, China) medium with 20 g/L urea at 30 °C overnight for pre-culture. Then pre-cultures were diluted 1:100 into 100 mL TSB medium with and without 20 g/L urea, respectively in 500-mL conical flasks. *E. coli, S. aureus*, and *B. subtilis* were cultivated in TSB medium with 20 g/L urea (pH ≈ 7.4) at 37 °C.

### Optical density measurement

The growth of *S. pasteurii* cultivated in TSB medium with 20 g/L urea and no urea respectively, was monitored in standard quartz cells by measuring the optical density at OD 600 nm with a UV-2600 UV–Vis spectrophotometer (SHIMADZU, Japan). A standard quartz cell contains 1 mL solution. When their OD_600_ was > 1, the test samples were diluted with sterile water with proper dilution factors for OD measurement to make sure OD_600_ is below 1. And the actual values of OD_600_ were obtained by multiplying the dilution factors.

### Zeta potential measurement

*Sporosarcina pasteurii, E. coli*, and *B. subtilis*, respectively were grown in TSB containing 20 g/L urea. When OD_600_ was about 1, the cell cultures were collected and centrifuged at 4200×*g* for 5 min. After discarding the supernatant, the cell pellets were washed with PBS three times to remove medium residuals. The bacterial aggregate was resuspended in 1 mL PBS, 1 mL calcium chloride solution (0.2 mol/L) and 1 mL calcium chloride solution (2 mol/L) respectively to obtain three kinds of bacterial suspensions (OD_600_ ≈ 1), and then was transferred to Malvern polystyrene U-shaped cell individually. Zeta potentials of these samples were measured with a Zetasizer Nano ZS instrument (Malvern Instruments, United Kingdom). The values presented are the average of three test results, and the standard deviation was considered as the error range.

### Scanning electron microscope (SEM) characterization

*Sporosarcina pasteurii*, *B. subtilis* and *E. coli* were cultivated in TSB medium with 20 g/L urea until OD_600_ was about 1. One milliliter bacterial suspension was collected and treated with 0.5 mL calcium chloride solution (1 mol/L), or calcium chloride solution (1 mol/L) and urea solution (1 mol/L) for 30 min, respectively. The untreated bacterial suspensions were set as controls. The samples were centrifuged at 4200×*g* for 5 min to obtain precipitation. After washing with sterile water three times, the precipitation was fixed in 2.5% glutaraldehyde solution for 4 h and then washed again to remove the residual glutaraldehyde. The samples were dehydrated with gradient dehydration by ethanol (30%, 50%, 70%, 85%, 95% and 100%) for 20 min for each concentration. Then the diluted samples were loaded on the clean silicon wafer and left to dry. The images of the samples were obtained using a Zeiss Ultra Plus-43-12 FESEM (Zeiss, Germany). The energy dispersive spectrometer (EDS) results were evaluated with the electron probe configured in the FESEM for elemental analysis.

### Intracellular urease activity and mineralization precipitation

The intracellular urease activity was determined by measuring the amount of ammonia released from urea. Firstly, the urease of *S. pasteurii* was extracted by Bacterial Protein Extraction Kit (BBI Life Sciences Corporation). Then 0.1 mol/L urea was added into the same volume of extracted urease solution for 30 min at 37 °C. The amount of ammonia released from urea was measured using phenol-hypochlorite assay method [41]. One unit of urease is defined as the amount of enzyme hydrolyzing urea to produce one micromole ammonia per minute. The specific urease activity (IU/OD) was obtained by dividing the urease activity (IU) by the bacterial biomass (OD_600_).

Two milliliter bacterial suspension of *S. pasteurii* under different conditions as indicated in the text was collected, and mixed with 1 mL urea (1 mol/L) and 1 mL CaCl_2_ (1 mol/L) for 15 min to produce the precipitation. Precipitation mass was obtained by centrifugation (8000 rpm, 5 min) and then dried at 50 °C for 8 h before being weighed.

### Whole genome sequencing

Sequencing was performed at Genewiz Bioinformatics Technology Co., Ltd (Suzhou, China). Genomic DNA was sheared, and then 20 Kb double-stranded DNA fragments was selected. DNA fragments were end repaired and ligated with universal hairpin adapters. Subsequently, the SMRTbell library was prepared and sequenced in PacBio Single Molecule Real-Time (SMRT) instrument. Then the PacBio reads were assembled using SmrtLink. The prodigal (Prokaryotic Dynamic Programming Genefinding Algorithm) software has been used for finding coding genes in bacteria. The coding genes were annotated with National Center for Biotechnology Information (NCBI) nr database by Diamond. The proteins encoded by genes were classified on a phylogenetic classification by the database of COG (Clusters of Orthologous Groups). The corresponding fastq files of *S. pasteurii* was deposited into NCBI SRA database with the NCBI Accession Number: SRR8727707.

### Transcriptomics analysis

The total RNA of samples were extracted with the TRIzol Reagent (Invitrogen, USA)/RNeasy Mini Kit (Qiagen, Germany). Total RNA of each sample was quantified and qualified by Agilent 2100 Bioanalyzer (Agilent Technologies, USA), NanoDrop (Thermo Fisher Scientific Inc., USA) and 1% agarose gel. Illumina RNA sequencing was carried out by Genewiz following their standard analysis method. Gene FPKMs were computed by summing the FPKMs of the transcripts in each gene group with Htseq (V 0.6.1). FPKM represents “expected number of fragments per kilobase of transcript sequence per millions base pairs sequenced”, which is calculated on the basis of the length of the fragments and the reads count mapped to each fragment. Genes with the absolute value of log_2_ (fold change) ≥ 1 and the corrected p-values less than 0.05 were assigned as significantly differentially expressed genes (DEGs).

## Supplementary information

**Additional file 1.** The list of coding genes and locations of *S. pasteurii*.

**Additional file 2.** The list of classification of genes (COG) of *S. pasteurii*.

**Additional file 3: Table S1.** Detailed data of genes regulation and location which were related to ATPase and urease. **Table S2.** DEGs related to ribosome. **Table S3.** EDS analysis of lumpy substances in Fig. 5. **Table S4.** DEGs related to four enzymes of fatty acid degradation.

**Additional file 4.** The list of the significant differential expression genes of SP2 VS SP1 and SP3 VS SP1.

## Data Availability

The materials and datasets for the current study are available from the corresponding author on reasonable request.

## Abbreviations

MICP: microbiologically induced calcite precipitation
TSB: trypticase soy broth
SEM: scanning electron microscopy
EDS: energy dispersive spectrometer
OD: optical density
COG: classification of genes
SMRT: single molecule real-time
FPKMs: fragments per kilobase of transcript sequence per millions base pairs sequenced
DEGs: differentially expressed genes
log_2_ (fold change): the log base 2 of the variation
padj: p-value adjusted by false discovery rate
