# Supplementary material for: Beneficial factors for biomineralization by ureolytic bacterium Sporosarcina pasteurii
Source: Microb Cell Fact. 2020 Jan 23;19:12. doi: 10.1186/s12934-020-1281-z (PMC6979283; doi:10.1186/s12934-020-1281-z)
Supplement: Supplementary file 3 — Additional file 3: Table S1. Detailed data of genes regulation and location which were related to ATPase and urease. Table S2. DEGs related to ribosome. Table S3. EDS analysis of lumpy substances in Fig. 5. Table S4. DEGs related to four enzymes of fatty acid degradation. [file 12934_2020_1281_MOESM3_ESM.docx]

**Additional file 3**

**Beneficial Factors for Biomineralization by Ureolytic Bacterium** ***Sporosarcina pasteurii***

Liang Ma^1^, Ai-Ping Pang^1^, Yongsheng Luo^1^, Xiaolin Lu^1#^, and Fengming Lin^1#^

^1^State Key Laboratory of Bioelectronics, School of Biological Science and Medical Engineering, Southeast University, Nanjing 210096, Jiangsu Province, P. R. China

^#^Corresponding author:

Fengming Lin

E-mail address: linfengming@seu.edu.cn

Xiaolin Lu

E-mail address: [lxl@seu.edu.cn](mailto:lxl@seu.edu.cn)

Author’s E-mail address:

Liang Ma [220184922@seu.edu.cn](mailto:220184922@seu.edu.cn)

Ai-ping Pang applepangtju@126.com

Yongsheng Luo 230189586@seu.edu.cn

Table S1. Detailed data of genes regulation and location which were related to ATPase and urease. The value was got by comparing the transcription level of *S. pasteurii* without urea to that with urea. The criteria for significant differences of gene: log_2_ (fold change) >= 1 and padj <= 0.05.

| Gene ID^1^ | Gene Name | log_2_ (fold change)^2^  (no urea / urea) | padj^3^ | log_2_ (fold change)^2^  (MICP treated before/after) | padj | from | to |
| --- | --- | --- | --- | --- | --- | --- | --- |
| 1_2285 | atpC | 1.50 | 2.67E-12 | 1.45 | 1.50E-09 | 2396395 | 2396799 |
| 1_2286 | atpD | 1.81 | 7.11E-15 | 1.27 | 1.81E-05 | 2396918 | 2398333 |
| 1_2287 | atpG | 2.25 | 4.53E-27 | 1.94 | 4.16E-15 | 2398526 | 2399383 |
| 1_2288 | atpA | 2.02 | 5.42E-23 | 1.95 | 5.76E-16 | 2399517 | 2401025 |
| 1_2289 | atpH | 2.06 | 4.19E-25 | 2.34 | 4.33E-25 | 2401053 | 2401592 |
| 1_2290 | atpF | 1.58 | 4.71E-13 | 2.10 | 1.18E-19 | 2401589 | 2402143 |
| 1_2291 | atpE | 1.69 | 1.88E-14 | 2.00 | 2.78E-17 | 2402327 | 2402539 |
| 1_2292 | atpB | 1.39 | 4.23E-08 | 1.41 | 5.09E-08 | 2402604 | 2403311 |
| 1_2293 | atpI | 2.01 | 7.49E-12 | 2.55 | 1.40E-35 | 2403436 | 2403807 |
| 1_611 | UreA | -0.07 | 0.76 | -0.73 | 0.000109305 | 636643 | 636945 |
| 1_612 | UreB | 0.73 | 0.0016 | -0.21 | 0.409760236 | 636997 | 637377 |
| 1_613 | UreC | 1.25 | 2.85E-11 | -0.85 | 7.41E-05 | 637381 | 639093 |
| 1_614 | UreE | 1.74 | 8.30E-19 | -0.26 | 0.190583216 | 639169 | 639612 |
| 1_615 | UreF | 1.48 | 5.89E-13 | -0.39 | 0.129537725 | 639554 | 640294 |
| 1_616 | UreG | 1.41 | 3.01E-11 | -0.37 | 0.154987881 | 640505 | 641140 |
| 1_617 | UreD | 1.58 | 2.09E-15 | -0.22 | 0.469122695 | 641115 | 641942 |

^1^ Gene ID is the serial number of genes in the sequencing process.

^2^ log_2_ (fold change): the log base 2 of the variation

^3^ padj: p-value adjusted by FDR (false discovery rate)

Table S2. DEGs related to ribosome. The value was got by comparing the transcription level of *S. pasteurii* without urea to that with urea.

| Gene ID | Gene Name | log_2_ (fold change)  (urea / no urea) | padj |
| --- | --- | --- | --- |
| 1_458 | RPL7Ae | -1.89 | 1.83E-21 |
| 1_2817 | MRPL23 | 2.01 | 2.44E-18 |
| 1_2819 | RPS19 | 2.02 | 9.01E-18 |
| 1_2820 | MRPL22 | 2.14 | 3.99E-17 |
| 1_2822 | MRPL16 | 1.63 | 6.33E-15 |
| 1_2818 | MRPL2 | 1.71 | 1.17E-14 |
| 1_2824 | MRPS17 | 1.77 | 1.78E-14 |
| 1_956 | MRPL21 | 2.10 | 3.04E-14 |
| 1_2662 | MRPL9 | -1.67 | 3.64E-14 |
| 1_2821 | RPS3 | 1.76 | 3.72E-14 |
| 1_2828 | MRPS14 | 1.69 | 9.59E-14 |
| 1_2827 | MRPL5 | 1.59 | 1.07E-13 |
| 1_2802 | MRPL1 | 1.97 | 2.16E-13 |
| 1_2659 | MRPS18 | 1.41 | 8.08E-13 |
| 1_1216 | MRPL33 | 1.83 | 1.75E-12 |
| 1_1726 | MRPL19 | 2.19 | 4.62E-12 |
| 1_2826 | MRPL24 | 1.61 | 1.68E-11 |
| 1_2803 | MRPL10 | 1.73 | 3.25E-11 |
| 1_2829 | RPS8 | 1.39 | 1.32E-10 |
| 1_2814 | MRPS10 | 2.07 | 1.38E-10 |
| 1_2825 | MRPL14 | 1.46 | 4.46E-10 |
| 1_2816 | MRPL4 | 1.57 | 7.14E-10 |
| 1_1678 | MRPS2 | 1.50 | 8.30E-10 |
| 1_2823 | RPL29 | 1.86 | 1.45E-09 |
| 1_958 | MRPL27 | 1.53 | 3.52E-09 |
| 1_2810 | MRPS7 | 1.44 | 4.65E-09 |
| 1_2815 | MRPL3 | 1.64 | 5.99E-09 |
| 1_2830 | MRPL6 | 1.13 | 7.45E-08 |
| 1_2833 | MRPL30 | 1.01 | 1.11E-07 |
| 1_2804 | MRPL12 | 1.47 | 1.33E-07 |
| 1_2808 | RPL7A | 1.69 | 2.32E-07 |
| 1_541 | MRPL35 | 1.74 | 3.45E-07 |
| 1_2657 | MRPS6 | 1.53 | 4.88E-07 |
| 1_1274 | RPS20 | 1.45 | 6.64E-07 |
| 1_1659 | MRPS15 | 1.35 | 7.67E-07 |
| 1_2831 | MRPL18 | 1.08 | 9.36E-07 |
| 1_1746 | MRPL28 | 1.52 | 1.11E-06 |
| 1_1730 | MRPS16 | 1.17 | 1.63E-06 |
| 1_2809 | MRPS12 | 1.28 | 6.61E-06 |
| 1_2839 | RPS13 | 1.11 | 1.23E-05 |
| 1_2838 | MRPL36 | 1.05 | 0.000262 |
| 1_2801 | MRPL11 | 1.03 | 0.000663 |
| 1_542 | MRPL20 | 1.81 | 0.002195 |

Table S3. EDS analysis of lumpy substances in Fig. 5.

| Element | Weight (%) | Atom (%) |
| --- | --- | --- |
| C | 18.26 | 26.86 |
| O | 55.84 | 61.67 |
| P | 0.36 | 0.21 |
| Ca | 25.54 | 11.26 |
| Total | 100.00 | 100.00 |

Table S4. DEGs related to four enzymes of fatty acid degradation.

| Enzyme ID | Enzyme Name | Related Gene ID | log_2_ (fold change)  (MICP before/after) | padj |
| --- | --- | --- | --- | --- |
| EC 6.2.1.3 | long-chain acyl-CoA syntetase | 1_1977 | -1.39 | 5.71E-07 |
|  |  | 1_679 | -2.99 | 2.52E-23 |
| EC 1.3.8.7 | acyl-CoA dehydrogenase | 1_673 | -2.22 | 7.92E-19 |
|  |  | 1_680 | -3.33 | 1.77E-18 |
|  |  | 1_438 | -1.11 | 1.83E-05 |
| EC 1.1.1.35 | 3-hydroxyacyl-CoA dehydrogenase | 1_221 | -1.38 | 1.71E-10 |
| EC 2.3.1.16 | acetyl-CoA C-acetyltransferase | 1_687 | -2.69 | 3.26E-19 |
|  |  | 1_2322 | -1.46 | 1.38E-13 |
|  |  | 1_684 | -2.59 | 6.63E-12 |
|  |  | 1_437 | -1.51 | 1.69E-09 |
|  |  | 1_222 | -1.42 | 3.15E-08 |
